# TAILORED INTERVENTION PROGRAM USING HUMANOID AI MINI-ROBOT FOR OLDER ADULTS WITH DEPRESSION IN RURAL AREAS

**DOI:** 10.1093/geroni/igad104.2447

**Published:** 2023-12-21

**Authors:** Young Mi Lim, Jungsug Kim, Junghee Lim, Minjung Sun

**Affiliations:** Yonsei University Wonju College of Nursing, Wonju, Republic of Korea; Yeoju Institute of Technology, Yeonju, Republic of Korea; Yeoju Community Mental Health Welfare Center &Suicide Prevention Center, Yeoju, Republic of Korea; Kyung Hee University, Seoul, Republic of Korea

## Abstract

Loneliness, and social isolation have worsened because most mental health centers were forced to suspend elderly-centric programs due to COVID-19. Recently, with the demand for a frail elderly-care model that enables non face-to-face interactions, strategies to utilize various technologies using robots for depression prevention are emerging. This pilot study aimed to develop and analyze a tailored intervention program using humanoid AI mini-robot for older adults with depression in rural areas. An experimental group of eight depressed elders participated daily in a tailored intervention program using humanoid AI mini-robot involving physical (stretching exercise) and emotional activities (listening to music) for ten weeks. A control group of ten depressed older adults received routine case management with non face-to-face at a mental health center. We compared the grip strengths and levels of depression and mental well-being between the experimental and control groups before and after the intervention, using Mann-Whitney U test and Wilcoxon signed rank test Statistically significant difference (U =12.0, p < 0.05) was observed between the mental well-being levels of the two groups; by contrast, grip strengths and depression levels did not differ significantly. The experimental group exhibited a significant difference (Z = –2.038, p < 0.05) between pre- and post-intervention grip strengths. The number of subjects was too small and the intervention period was not long enough to draw a meaningful conclusion. However, the findings have implications for the need of potential tailored interventions using humanoid AI mini-robot for mental well-being of community dwelling frail older adults.

